# Photo-Induced Antitumor Effect of 3,6-Bis(1-methyl-4-vinylpyridinium) Carbazole Diiodide

**DOI:** 10.1155/2013/930281

**Published:** 2012-12-27

**Authors:** Ya-Shuan Chou, Cheng-Chung Chang, Ta-Chau Chang, Tsung-Lin Yang, Tai-Horng Young, Pei-Jen Lou

**Affiliations:** ^1^Institute of Biomedical Engineering, College of Medicine and College of Engineering, National Taiwan University, Taipei 10002, Taiwan; ^2^Graduate Institute of Biomedical Engineering, National Chung Hsing University, Taichung 40254, Taiwan; ^3^Institute of Atomic and Molecular Sciences, Academia Sinica, Taipei 10617, Taiwan; ^4^Department of Otolaryngology, National Taiwan University Hospital and College of Medicine, Taipei 10002, Taiwan

## Abstract

We have applied a fluorescent molecule 3,6-bis(1-methyl-4-vinylpyridinium) carbazole diiodide (BMVC) for tumor targeting and treatment. In this study, we investigated the photo-induced antitumor effect of BMVC. *In vitro* cell line studies showed that BMVC significantly killed TC-1 tumor cells at light dose greater than 40 J/cm^2^. The fluorescence of BMVC in the tumor peaked at 3 hours and then gradually decreased to reach the control level after 24 hours. *In vivo* tumor treatment studies showed BMVC plus light irradiation (iPDT) significantly inhibited the tumor growth. At day 24 after tumor implantation, tumor volume was measured to be 225 ± 79 mm^3^, 2542 ± 181 mm^3^, 1533 ± 766 mm^3^, and 1317 ± 108 mm^3^ in the iPDT, control, light-only, and BMVC-only groups, respectively. Immunohistochemistry studies showed the microvascular density was significantly lower in the iPDT group. Taken together, our results demonstrated that BMVC may be a potent tumor-specific photosensitizer (PS) for PDT.

## 1. Introduction 

Tumor-targeting therapy has emerged as an effective and attractive treatment for cancer. Among the various cancer-specific targets tested, telomerase has gathered much attention in recent years. Telomerase is detected in about 85% to 90% of cancer cells, but in a low level of normal cells [1]. The maintenance of telomere length by telomerase is required for unlimited proliferation of cancer cells. Telomere has been the target for the development of cancer probes, and telomerase inhibitors have been developed to inhibit telomerase activity and limiting cancer cell growth [2]. 

In the search for tumor-targeting agents, we have recently developed a fluorescent molecule 3,6-bis(1-methyl-4-vinylpyridinium) carbazole diiodide (BMVC) for recognizing specific quadruplex structures such as the T_2_AG_3_ telomeric repeats and inhibiting the telomerase activity [3–5]. Intriguingly, the fluorescence of BMVC detected in cancer cells was much stronger than that in normal cells, suggesting it to be a good candidate for a tumor-targeting agent [3]. The maximum absorption of BMVC is shifted from 435 to 460 nm and the fluorescence intensity increases significantly when BMVC interacts with DNA [5]. Because of the ability of telomerase inhibition, BMVC induces accelerated senescence of cancer cells [6]. 

Photodynamic therapy (PDT) is an effective treatment for cancerous and precancerous lesions [7]. The advantages of PDT are that it can be repeated in the same site if necessary, and it is less destructive than traditional surgery. PDT requires PSs that are activated by specific wavelengths of light. Illumination of tumor results in the destruction of cells due to a photochemical reaction. Reactive oxygen species, including singlet oxygen and free radicals, are generated by the photochemical reaction [8, 9]. This photochemical reaction is capable of inducing cellular apoptosis and necrosis, by evoking oxidative stress [10]. In addition, PDT may cause tumor cell death indirectly by damaging tumor-associated vasculature or activating host immune responses [9, 11]. 

Previously we have investigated the fluorescence resonance energy transfer (FRET) binary system that consists of BMVC conjugating porphyrin [12]. We found that PDT efficiency is greater when excited by 470 nm of light as compared to 510 nm of light. It is surprised that better PDT efficiency is observed upon exciting the FRET donor (BMVC) rather than the acceptor (porphyrin). This extra phototoxic effect could result from a type I photodynamic reaction [13–15] because we detected neither the characteristic spectral signal of singlet oxygen (1270 nm) from BMVC in D_2_O solution nor the decrease of 3-diphenylisobenzofuran (DPBF) signal in organic solvent. Here, we have examined the phototoxicity mechanism of BMVC and illustrated its potential to be used as a photosensitizer (PS) for photodynamic therapy (PDT).

Despite the potential advantages in clinical application, PDT has several limitations that hinder its wide clinical acceptance. Among them, sustained skin photosensitivity and low tumor selectivity are two major problems for the PSs [16]. The purpose of this study was to investigate the photochemical effects of BMVC on tumor cells. Cellular cytotoxicity of BMVC was evaluated in TC-1 cell line. The antitumor effect of BMVC combined with a specific wavelength of light was investigated in the animal model. 

## 2. Materials and Methods

 BMVC was synthesized from 3,6-dibromocarbazole as described previously [17]. 

### 2.1. Cell Line

The mouse (C57BL/6, B6) lung tumor line TC-1 was maintained in a humidified 5% CO_2_ incubator at 37°C. TC-1 cells were grown in RPMI 1640 supplemented with 10% fetal calf serum (FCS), 50 U/mL penicillin, 50 U/mL streptomycin, and 0.4 mg/mL G418 [18]. 

### 2.2. Fluorescence Spectrum

Cells were treated with 20 *μ*M BMVC and then washed, trypsined, and resuspended. The cell suspensions were collected and measured using a fluorescence microplate reader (SpectraMax M2e, Molecular Devices, Sunnyvale, CA, USA) with 460 nm excitation wavelengths. Fluorescence emission wavelengths of BMVC at 500–750 nm were recorded by scanning 10 nm wavelength windows for each sample [19].

### 2.3. Cytotoxicity Assay

Cells were grown in 96-well plates (2000 cells/well) and then incubated with BMVC for 6 hours and changed to fresh culture medium (without phenol red), and the cytotoxicity was determined by the Alamar Blue assay [20]. 10% Alamar Blue in 200 *μ*L of culture media was added to each test well and analyzed spectrophotometrically at the absorbance difference between 570 and 600 nm. 

### 2.4. Free Radical Assay

The light source for measurement of PDT effect was a white light (a 200 W Xenon lamp that passes through a 400–700 nm mirror module) finally, the light power was 100 mW/cm^−2^ on the sample surface. 

### 2.5. Animal Study

All animal studies were approved by the Institutional Animal Care Committee. Male C57BL/6 (B6) mice, weighing 20–28 g, were supplied by the University Laboratory Animal Center and were allowed free access to food and water. Around 5 × 10^4^ TC-1 cells in 100 *μ*L of HBSS were injected s.c. into B6 mice. BMVC at concentration of 5 mg/kg was injected (single dose) i.p. after the tumor grew to around 50 to 100 mm^3^. The length (*L*) and width (*W*) of the TC-1 tumor mass and the body weight of mice at 1, 7, 10, and 14 days after BMVC injection were recorded. Tumors were measured in two orthogonal directions, and the tumor volumes were estimated as (*LW* ^2^)/2 [18, 21]. All animals challenged with TC-1 cells had developed a palpable tumor at day 10.

### 2.6. Distribution of BMVC in Mice

Mice were sacrificed at 1, 2, 3, 5, 12, 24, and 72 hours after BMVC injection, and the tissues were removed, homogenized, and analyzed by a fluorescence microplate reader (excitation wavelength at 460 nm). Tissues from mice 3 hours after BMVC injection were collected, washed with PBS, and then analyzed by the flow cytometry (FACS Calibur and CellQuest software, BD Biosciences). 

### 2.7. Photodynamic Therapy

#### 2.7.1. *In Vitro* Study

Cells were incubated with 5 *μ*M BMVC in darkness for 6 hours at 37°C. After wash with PBS, the cells were immediately exposed to different doses of light at 445 nm (LDM445, Pkebtunax, Germany). Cell toxicity was determined 24 hours after light treatment with energy doses of 1, 5, 10, 20, 40, 60, and 80 J/cm^2^. 

#### 2.7.2. *In Vivo* Study

BMVC (5 mg/kg) was injected to the mice when the size of tumor reached 0.8 cm in diameter (about 14 days after TC-1 injection). Three hours after BMVC injection, mice were subjected to interstitial light treatment as described before [22, 23]. In brief, a needle (21 G) was pushed percutaneously into the tumor, and the fiber was passed through the needle. The tip of the fiber was managed to be at about 1 mm outside of the needle tip to ensure it actually touched the tumor tissue. A single dose of 150 J was delivered to the tumor by a 445 nm diode laser (LDM445, Pkebtunax, Germany).

### 2.8. Immunohistochemistry

Cryosections of the tumor at 10 *μ*m thickness were fixed with 10% formalin, washed, and then immunostained for *α*-smooth muscle actin (*α*-SMA, 2 *μ*g/mL, DAKO, Glostrup, Denmark). The number of *α*-SMA positive cells was counted in three nonoverlapping regions. Unit counts were expressed as the number of *α*-SMA positive unit per mm^2^ of tumor tissue. 

### 2.9. Statistical Analysis

Student's *t*-test was used to evaluate the response to a change in conditions. Data were subjected to statistical analysis using the SPSS for Windows version 10. 

## 3. Results

### 3.1. BMVC Fluorescence Spectra

**Figure 1:**
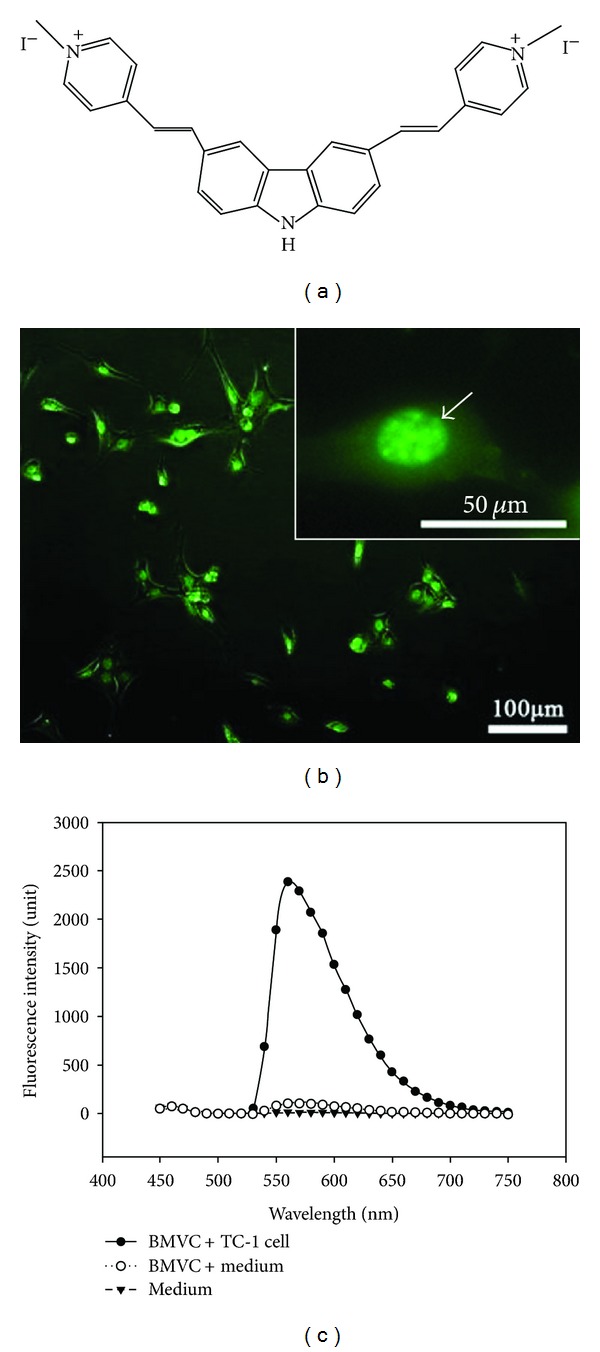
(a) Chemical structure of BMVC. (b) Fluorescence image of TC-1 cells incubated with 20 *μ*M of BMVC. (c) Fluorescence spectra of BMVC before and after incubation with TC-1 cells.

The chemical structure of BMVC was shown in Figure 1(a). When TC-1 cells were cultured in the presence of BMVC, the fluorescence signals were detected mainly in the cell nuclei (Figure 1(b)). Fluorescence emission spectra of BMVC were depicted in Figure 1(c). It clearly demonstrated that the fluorescence intensity of BMVC increased for almost 2 orders of magnitude upon interacting with cells. 

### 3.2. *In Vitro* Cytotoxicity and Phototoxicity

**Figure 2:**
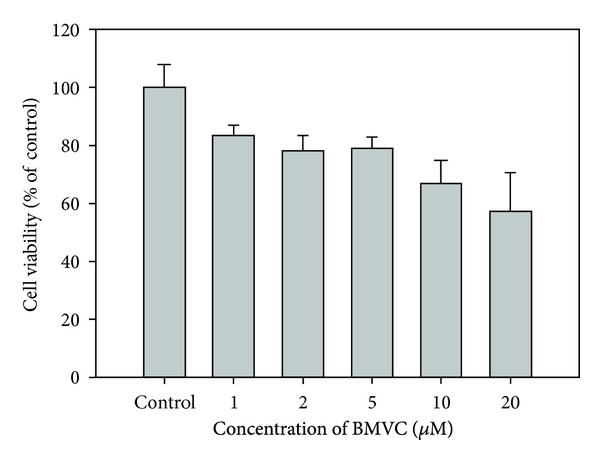
Cytotoxicity of BMVC on TC-1 cells. TC-1 cells were treated with different concentrations of BMVC for 24 hours and cell viability was determined by the Alamar Blue assay.

Cytotoxic effects of BMVC to TC-1 cells were evaluated. Low dose BMVC treatment did not have apparent toxicity to the cells. As shown in the Figure 2, ~80% of cells were still viable when the BMVC concentrations were below 5 *μ*M. When the BMVC concentrations were above this level, cytotoxicity effects became obvious. The cell viability decreased to ~60% when cells were treated with 20 *μ*M of BMVC. Since 5 *μ*M of BMVC treatment did not show significant cytotoxicity to the cells, we use this concentration for the subsequent PDT treatment. 

**Figure 3:**
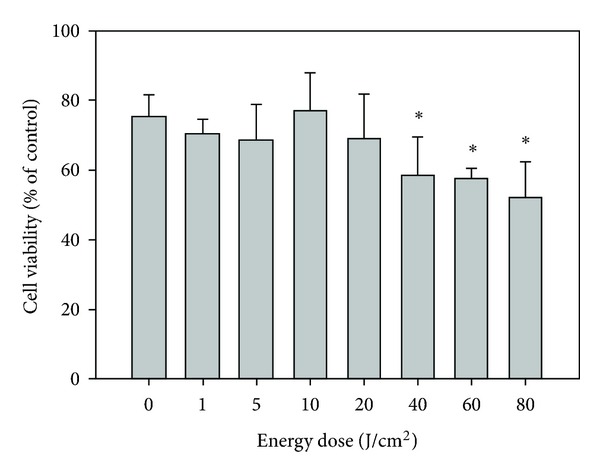
Photodynamic effect of BMVC. TC-1 cells were incubated with BMVC and then subjected to light treatment as described in Section 2. Cell viability was measured. *P < 0.05 as compared to the 0 J/cm^2^.

Photo-induced cytotoxicity of BMVC was evaluated as a function of light dose.  Figure 3 showed the photo-induced cytotoxicity of BMVC upon the treatment of 0, 1, 5, 10, 20, 40, 60, and 80 J/cm^2^ light doses in TC-1 cells. It was found that BMVC coupled with low light doses (below 20 J/cm^2^) did not show apparent cytotoxicity. However, cell survival of the BMVC coupled with higher light doses (40, 60, and 80 J/cm^2^) appreciably decreased to 66.9%, 57.5%, and 52.1%, respectively. 

**Figure 4:**
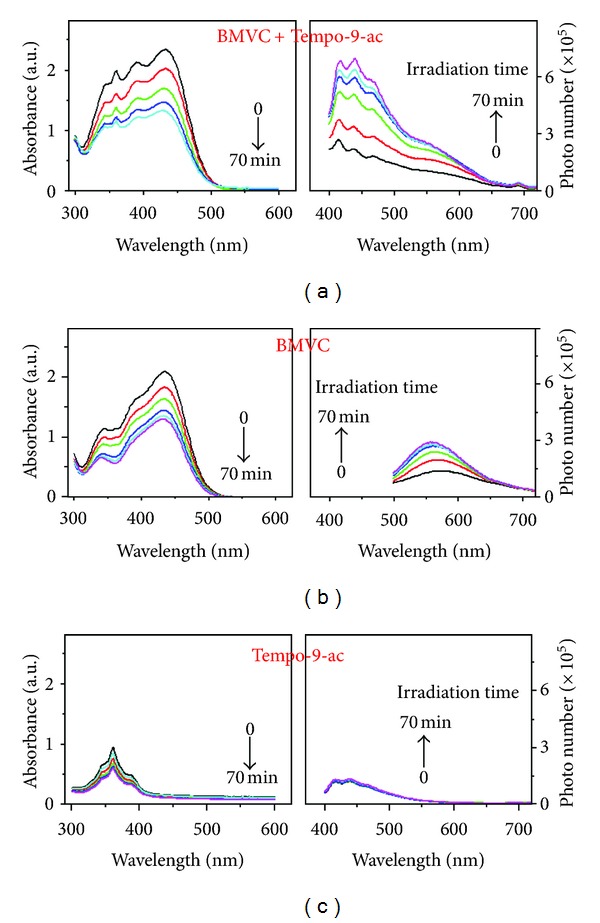
Absorption (left) and emission (right) spectra BMVC upon irradiation (400–700 nm 100 mW/cm^2^) with (a) and without (b) radical fluorescence probe TEMPO-9-ac. (c) Absorption and emission of Tempo-9-ac under similar irradiation condition. The excitation wavelength for emission spectra: 360 nm for (a) and (c), 440 nm for (b).

Type I sensitization assumes the formation of radicals via electron transfer (reductive or oxidative) involving the triplet state of the photosensitizer. The free radical probe 4-((9-acridinecarbonyl) amino)-2,2,6,6-tetramethylpiperidin-1-oxyl (TEMPO-9-ac, Invitrogen, Carlsbad, CA, USA) captures radicals (mostly long-lived carbon or sulfur centered) resulting in fluorescence turnon (*λ*ex/*λ*em = 360/440 nm) [24]. Irradiation of BMVC (10 **μ**M) generated apparent increase in signal from TEMPO-9-ac (10 **μ**M) (Figures 4(a) and 4(b)), compared to unchanged levels of the control. (Figure 4(c)). As the most type I cases [25, 26], Figure 4(a) also showed light-induced photo reduction of the BMVC, which was accompanied by bleaching, resulting in the long-range electron transfer through the water channel, followed by the superoxide generation [27]. This is why we cannot collect similar results as Figure 4 in DMSO solution (data not shown).

### 3.3. Distribution and Kinetics of BMVC in the Tumor and Normal Mice Tissues

**Figure 5:**
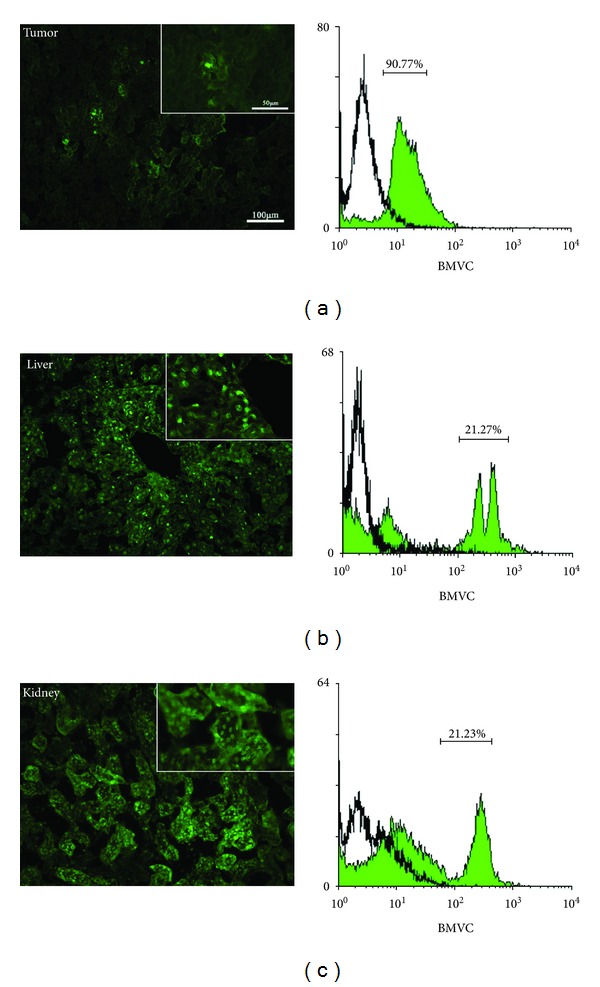
Distribution of BMVC in the tumor and normal mice tissues. Flow cytometry (left panel) and fluorescence images (right panel) of tumor (a), liver (b), and kidney (c) tissues were shown. The numbers in percentage shown in the left panel indicated the ratio of BMVC positive cells to the total number of cells.

**Figure 6:**
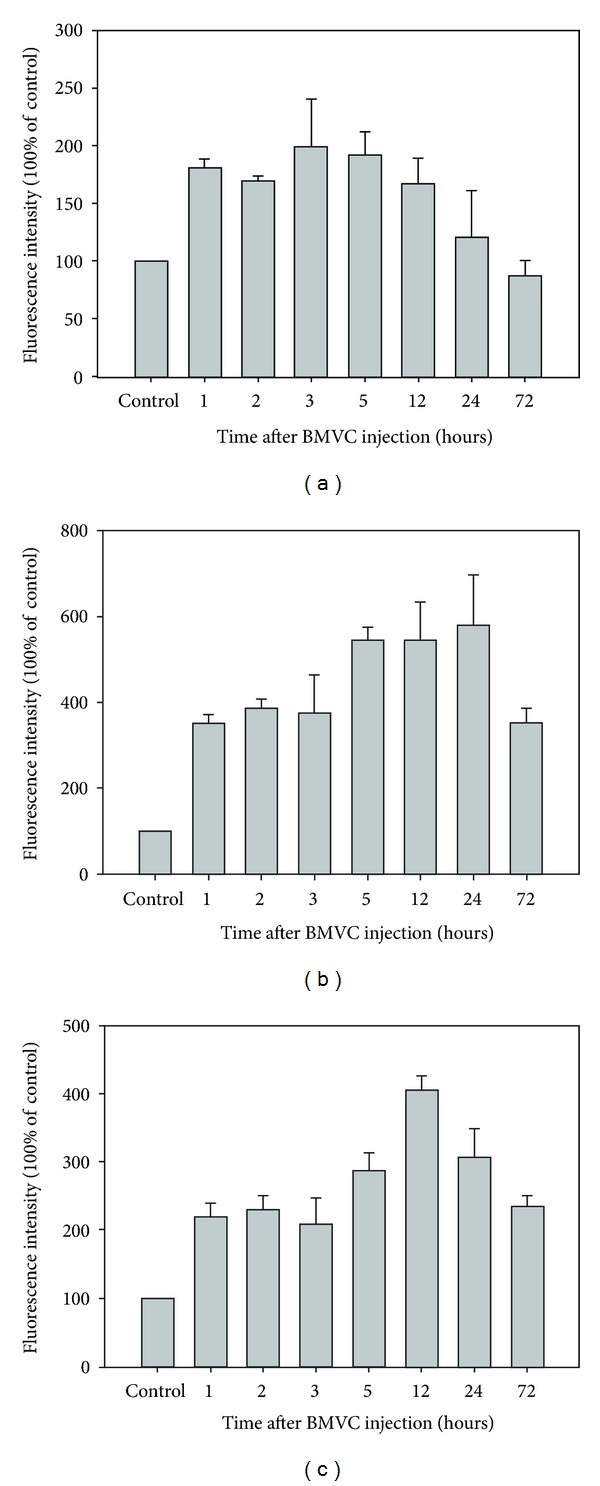
Kinetic of BMVC in the tumor and normal mice tissues. Mice were treated with BMVC and then sacrificed at different time points as described in Section 2. Fluorescence intensity of BMVC in the tumor (a), liver (b), and kidney (c) tissues was determined by the ELISA reader.

Liver and kidney are the main organs responsible for drug metabolism and excretion. We further measured the distribution of BMVC in the tumor, liver, and kidney tissues by flow cytometry and fluorescence microscopy at 3 hours after BMVC injection (Figure 5). The results showed that the majority (90.8%) of tumor cells were positive for BMVC fluorescence. On the other hand, only 47.2% of the liver cells and 30.9% of the kidney cells were positive for BMVC fluorescence, although the fluorescence intensity was stronger in liver and kidney than in the tumor. Kinetics of BMVC in the tumor and normal mice tissues were also investigated. After injection, the fluorescence of BMVC gradually increased in the tumor tissues, peaked at 3 hours, and then gradually decreased to reach the control level after 24 hours (Figure 6(a)). Similarly, the fluorescence of BMVC in the liver and kidney increased after drug injection, peaked at 24 and 12 hours, respectively, and then gradually decreased (Figures 6(b) and 6(c)). At 72 hours after injection, the BMVC fluorescence levels in the liver and kidney were still above the control level. However, the BMVC fluorescence in the brain, lung, and muscles showed no appreciable difference from the control tissues (data not shown). 

### 3.4. *In Vivo* Tumoricidal Effects

**Figure 7:**
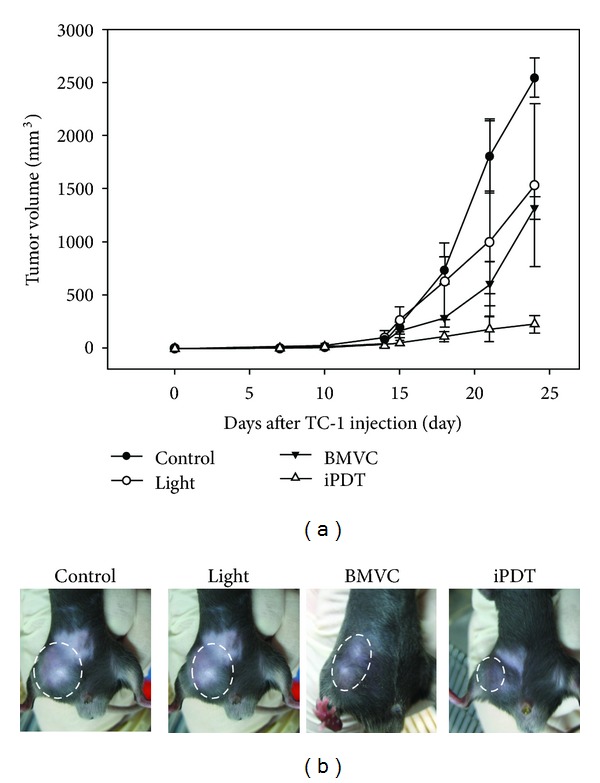
Antitumor effects of BMVC. Mice were transplanted with TC-1 cells and then subjected to light-alone, BMVC-alone, or BMVC plus light (iPDT) treatment as indicated. The changes in tumor volume (a) and pictures of tumors at 24 days posttumor transplantation (b) were shown. Arrow indicates the time point of treatment.

To evaluate the *in vivo* acute toxicity of BMVC in tumor-bearing mice, we injected TC-1 cells s.c. to B6 mice. After tumors grew to about 50 mm^3^, mice received i.v. injection of 5 mg/kg BMVC once. We then evaluated the effect of BMVC on tumor growth and body weight. The results showed no change in body weight between groups (data not shown), and there was no sign of acute toxicity in this BMVC dose.  Figure 7 showed the growth curves of tumors under different treatments. It clearly showed that BMVC plus light (iPDT) significantly inhibited the tumor growth *in vivo *(*P* < 0.05). At day 24 after tumor implantation, tumor volume was measured to be 225 ± 79 mm^3^, 2542 ± 181 mm^3^, 1533 ± 766 mm^3^, and 1317 ± 108 mm^3^, respectively, in the iPDT, control, light-only, and BMVC-only groups. There is only 6.76% evolution of tumor volume in iPDT group between day 14 and 24. Although the tumors in the light-only and the BMVC-only groups were also smaller than the control group, the difference did not reach statistically significant level. 

### 3.5. Immunohistochemistry

**Figure 8:**
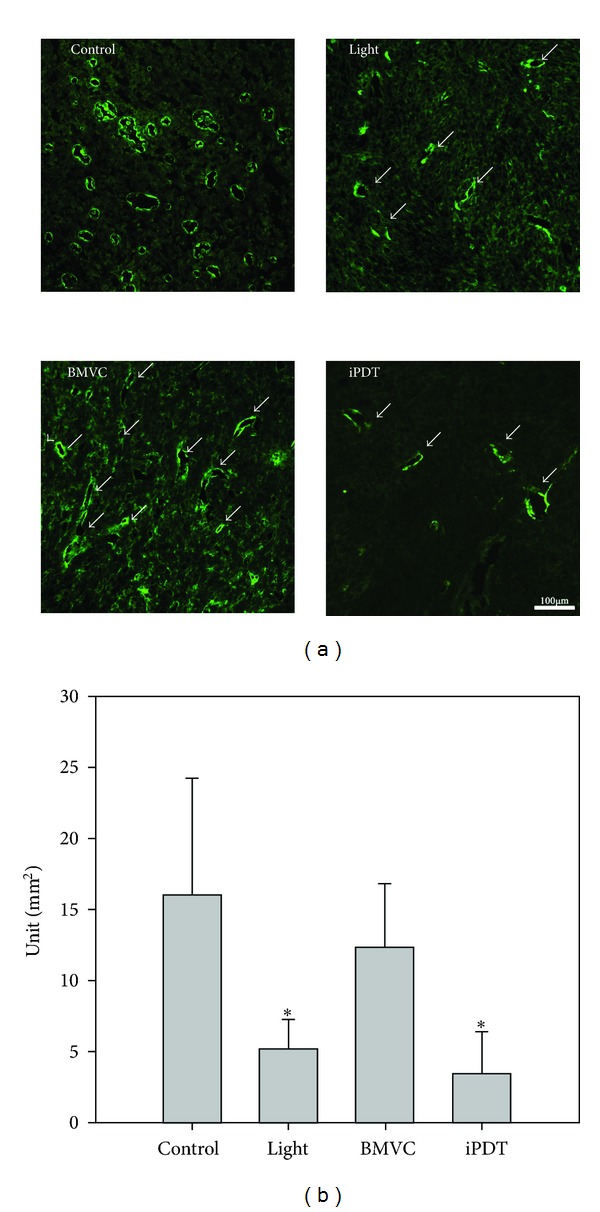
Microvascular densities of control and iPDT treated tumors. Immunohistochemical staining for *α*-SMA (a) and semiquantitative analyses results (b) were shown. *P < 0.05.

PDT is known to cause microvascular destruction in tumor tissues. The direct cytotoxic activity and microvascular damage contribute to the destruction of tumor [9, 28]. To investigate the microvascular density in the tumors, we measured *α*-smooth muscle actin (*α*-SMA) positive cells in different groups of tumors.  Figure 8 showed the quantification of *α*-SMA positive cells under different conditions. It is found that the microvascular density was significantly lower after both the light-only and the iPDT treatments. On the other hand, BMVC treatment showed minor effect on the microvascular density of tumors. 

## 4. Discussion

PDT has emerged as an effective means for cancer therapy. PSs are an essential element of PDT. In general, the photoactivation of the PS leads to an oxygen dependent oxidative-reaction resulting in cellular photodamage. The mechanisms of PDT involve direct oxidation of biological targets through hydrogen abstraction or electron transfer to yield radical chain reaction (type I reaction), as well as oxidation mediated by singlet oxygen (^1^O_2_) through energy transfer from triplets to molecular oxygen to initiate oxidative damage (type II reaction). In this study, we investigated the possibility of using BMVC as a PS for PDT. Illuminating TC-1 cells with 445 nm of light can result in the destruction of cells (Figures 2 and 3). Further experiments showed that BMVC plus light (iPDT) significantly inhibited the growth of tumor cells *in vivo* (Figure 7). These results suggest that BMVC is a useful PS for PDT.

Tumor-targeting therapy is the treatment of cancer cells without injuring the normal cells. However, one of the major drawbacks of current PSs is the lack of tumor selectivity. Many studies have been focused on the modification of specific porphyrin structures to achieve better tumor selectivity. Furthermore, the fluorescence of BMVC detected in cancer cells was much stronger than that in normal cells, letting it to be a good candidate for the tumor-targeting agent [3]. In this study, we showed that 90.8% of tumor cells were positive for BMVC fluorescence at 3 hours after i.v. injection (Figure 5(a)). On the other hand, only 47.2% of the liver cells and 30.9% of the kidney cells were positive for BMVC fluorescence (Figures 5(b) and 5(c)). Together, these results indicated that BMVC had certain degree of tumor selectivity and could be implemented as a tumor-targeting PS for PDT.

In addition to be used in the treatment of cancer, PSs were also implemented in the diagnosis of cancers. In 1924, red fluorescence of porphyrin was observed in experimental rat sarcomas by Policard [29]. This is the first observation of PS fluorescence in tumors. The followed experiments confirmed that some PSs accumulated in cancer cells and were possibly been used in cancer detection (photodynamic diagnosis, PDD) [30]. In PDD, fluorescence in cancer cells could be observed by either fluorescence spectroscopy or fluorescence imaging. Clinical application of PDD had been shown in premalignant oral tissue screening [31]. In this study, we noted that the fluorescence intensity of BMVC increased significantly upon interacting with tumor cells (Figure 1(c)). The emission spectrum was different between free BMVC molecules and BMVC plus TC-1 cells. These results were in line with our previous molecular chemical studies that showed strong fluorescence of BMVC that peaked at approximately 550 or 575 nm in the presence of DNA structure [5]. Since the fluorescence of BMVC significantly enhanced upon interacting with tumor cells and that 90.8% of *in vivo* tumor cells exhibited strong BMVC fluorescence, BMVC could be used as a potential molecule for PDD of cancer cells.

Although BMVC-PDT is effective *in vitro*, there are some limitations in the *in vivo* animal studies. The absorption wavelength (445 nm) of BMVC is insufficient to pass through the whole bulk of tumor cells. To overcome this problem, we used fine-needle interstitial light irradiation to shine the light into tumor tissues. This fine-needle interstitial PDT (iPDT) which combines with the BMVC treatment significantly inhibited tumor growth (Figure 7). There were 2 possible mechanisms to explain this significant tumor inhibition. One is the direct effect of BMVC on the tumor telomeres. As reported in our previous studies, BMVC was able to suppress the telomerase activity and induce senescence of cancer cells [3, 4]. Tumor formation and progression can be suppressed by BMVC treatment alone. The other is the effect of PDT may destruct the intratumoral vasculature and result in tumor ischemia and death by decreases in perfusion [21]. Our immunohistochemistry results in this study confirmed the destruction of intratumoral vasculature by BMVC-PDT (Figure 8). Intriguingly, tumors receiving light irradiation alone also showed significant reduction of intratumoral microvascular density. The thermal effect might decrease the vascular density in light irradiation alone group because we treated the mice by a high energy direct treatment. The mild thermal therapy has the ability to change vascular perfusion and oxygen supplied within the tumor microenvironment [32]. Nevertheless, the iPDT group has the smallest tumor volume certainly. In our study, we showed the photochemical effect occurred by BMVC treated combined with light. The mechanism behind this light induced microvascular destruction remains elusive and needs further investigation.

## 5. Conclusions

 We described a tumor-targeting therapy for the application of BMVC. In summary, the distinct properties of this fluorescent molecule provide a design of PS for PDT treatment. PDT experiments showed that BMVC-PDT significantly inhibited the growth of tumor cells both in the *in vitro* and *in vivo* studies. Our results demonstrated that BMVC may be a potent tumor-specific PS for PDT. A number of potential refinements can be incorporated into future studies based on this effect of BMVC, such as conjugate with other PSs to increase phototoxic effects and pi-conjugation lengths, which induce the bathochromic shift to enhance the penetration depth of the PDT laser. 

## Abbreviations

BMVC: 3,6-Bis(1-methyl-4-vinylpyridinium) carbazole diiodide
DPBF: 3-Diphenylisobenzofuran
FCS: Fetal calf serum
FRET: Fluorescence resonance energy transfer
PDD: Photodynamic diagnosis
PDT: Photodynamic therapy
PS: Photosensitizer.
